# Total Pancreatectomy with Islet Autotransplantation (TPIAT) as a Technique to Treat Chronic Idiopathic Pancreatitis: Early Experience Analysis

**DOI:** 10.17691/stm2024.16.6.05

**Published:** 2024-12-27

**Authors:** V.E. Zagainov, D.M. Kuchin, A.V. Kashina, L.A. Lugovaya, N.V. Zarechnova, T.A. Galanina, N.U. Naraliev, Y.I. Kolesnik, E.A. Vasilchikova, P.S. Ermakova, E.M. Zagaynov, Yu.A. Kucheryavy, A.Yu. Bogomolova, A.L. Potapov, I.Yu. Shirokova

**Affiliations:** MD, DSc, Director of the Institute of Surgery and Oncology; Privolzhsky Research Medical University, 10/1 Minin and Pozharsky Square, Nizhny Novgorod, 603005, Russia; Head of the Department of Faculty Surgery and Transplantology; Privolzhsky Research Medical University, 10/1 Minin and Pozharsky Square, Nizhny Novgorod, 603005, Russia; Deputy Chief Physician for Science and Innovation; Nizhny Novgorod Regional Oncologic Dispensary, 11/1 Delovaya St., Nizhny Novgorod, 603093, Russia; MD, PhD, Associate Professor, Department of Faculty Surgery and Transplantology; Privolzhsky Research Medical University, 10/1 Minin and Pozharsky Square, Nizhny Novgorod, 603005, Russia; Head of the Center for Abdominal Surgery; Nizhny Novgorod Regional Clinical Hospital named after N.A. Semashko, 190 Rodionova St., Nizhny Novgorod, 603093, Russia; PhD, Head of the Regenerative Medicine Laboratory, Research Institute of Experimental Oncology and Biomedical Technologies; Privolzhsky Research Medical University, 10/1 Minin and Pozharsky Square, Nizhny Novgorod, 603005, Russia; MD, PhD, Associate Professor, Department of Endocrinology and Internal Medicine; Privolzhsky Research Medical University, 10/1 Minin and Pozharsky Square, Nizhny Novgorod, 603005, Russia; MD, PhD, Head of the Anesthesiology and Resuscitation Center; Nizhny Novgorod Regional Oncologic Dispensary, 11/1 Delovaya St., Nizhny Novgorod, 603093, Russia; Assistant, Department of Anesthesiology, Resuscitation and Transfusiology; Privolzhsky Research Medical University, 10/1 Minin and Pozharsky Square, Nizhny Novgorod, 603005, Russia; Head of the Department of Resuscitation and Intensive Care for Patients with Combined Trauma; Nizhny Novgorod Regional Clinical Hospital named after N.A. Semashko, 190 Rodionova St., Nizhny Novgorod, 603093, Russia; Assistant, Department of Faculty Surgery and Transplantology; Privolzhsky Research Medical University, 10/1 Minin and Pozharsky Square, Nizhny Novgorod, 603005, Russia; Assistant, Department of Faculty Surgery and Transplantology; Privolzhsky Research Medical University, 10/1 Minin and Pozharsky Square, Nizhny Novgorod, 603005, Russia; Laboratory Assistant, Regenerative Medicine Laboratory, Research Institute of Experimental Oncology and Biomedical Technologies; Privolzhsky Research Medical University, 10/1 Minin and Pozharsky Square, Nizhny Novgorod, 603005, Russia; Laboratory Assistant, Regenerative Medicine Laboratory, Research Institute of Experimental Oncology and Biomedical Technologies; Privolzhsky Research Medical University, 10/1 Minin and Pozharsky Square, Nizhny Novgorod, 603005, Russia; Specialist in X-ray Endovascular Diagnostics and Treatment, Clinical Hospital No.1; Privolzhsky District Medical Center of Federal Medico-Biologic Agency of Russia, 14 Ilyinskaya St., Nizhny Novgorod, 603109, Russia; MD, PhD, Associate Professor, Head of the Gastroenterology Department; Ilyinskaya Hospital, M-9, 26^th^ km, Krasnogorsk, Moscow Region, 143421, Russia; Laboratory Assistant, Regenerative Medicine Laboratory, Research Institute of Experimental Oncology and Biomedical Technologies; Privolzhsky Research Medical University, 10/1 Minin and Pozharsky Square, Nizhny Novgorod, 603005, Russia; Laboratory Assistant, Optical Coherence Tomography Laboratory, Research Institute of Experimental Oncology and Biomedical Technologies; Privolzhsky Research Medical University, 10/1 Minin and Pozharsky Square, Nizhny Novgorod, 603005, Russia; PhD, Head of the Laboratory Research Department, Research Institute of Preventive Medicine; Privolzhsky Research Medical University, 10/1 Minin and Pozharsky Square, Nizhny Novgorod, 603005, Russia

**Keywords:** chronic pancreatitis, the *SPINK1* and *PRSS1* gene mutations, pancreas, pancreatectomy, islets of Langerhans, pancreatogenic diabetes

## Abstract

Chronic pancreatitis (CP) is a quite common disease with an annual incidence of up to 30 new cases per 100,000 persons in Russia, and 9.6 cases per 100,000 persons abroad. Idiopathic pancreatitis associated with genetic mutations ranks second in frequency. When conservative therapy is ineffective, surgical intervention is considered. Total pancreatectomy reduces pain, but leads to pancreatogenic diabetes mellitus. In case of hereditary pancreatitis caused by mutations in the *PRSS1*, *CFTR*, *SPINK1* genes, pancreatectomy becomes advisable, including for prevention of pancreatic cancer.

Isolation of Langerhans islets from the excised pancreas and their autotransplantation (total pancreatectomy with islet autotransplantation, TPIAT) provides additional treatment options. Such surgical interventions are not performed in Russia as of yet.

**The aim of the study** was to assess the effectiveness of total pancreatectomy followed by restoration of glucose tolerance in treatment of patients with chronic genetically determined pain pancreatitis.

**Materials and Methods:**

Two patients with chronic pain pancreatitis with the *SPINK1* and *PRSS1* genetic mutations were examined and underwent surgical total pancreatectomy. Islets were isolated from the excised glands and implanted into the liver. Postoperative followup included an assessment of quality of life and pain intensity based on questionnaires, as well as determination of the glycemic level.

**Results:**

Following total pancreatoduodenectomy and autotransplantation, a significant decrease in pain and an improvement in quality of life were noted. Transplanted islets’ function was reduced, due to their insufficient number, which required administration of exogenous insulin.

**Conclusion:**

The described experience demonstrates the TPIAT effectiveness in treatment of chronic pancreatitis, which can become a basis for further research and introduction of the technique into domestic clinical practice.

## Introduction

The annual incidence of chronic pancreatitis (CP) in Russia is up to 30 new cases per 100,000 persons [1], whereas as globally — 9.6 cases per 100,000 persons [2]. Idiopathic pancreatitis ranks second in frequency and is mainly genetically related as it is associated with mutations in the *PRSS1*, *CFTR*, *SPINK1*, and *CTRC* genes [3, 4].

Complex medical therapy of patients with CP is often ineffective. In particularly severe cases, one has to use opioid drugs, which, with long-term administration, lead to serious adverse effects [5]. According to modern clinical guidelines, when conservative therapy is ineffective, surgical treatment is considered. Resection or drainage interventions are not indicated if there is no dilation of the main pancreatic duct and in case of total fibrosis of the pancreatic tissue with no focal pathologies [6, 7]. Total pancreatectomy here effectively reduces the level of pain, but leads to pancreatogenic diabetes mellitus. A surgeon must have sufficient grounds to go in for such operation.

Pancreatectomy becomes advisable in case of proven idiopathic hereditary pancreatitis and identified mutations in the *PRSS1*, *CFTR*, *SPINK1*, or *CTRC* genes, especially when there is a risk of ductal adenocarcinoma [8, 9].

Isolation of islets of Langerhans (IL) from the excised pancreas and their subsequent injection to a patient has significantly expanded pancreatectomy prospects. Total pancreatectomy with islet autotransplantation (TPIAT) is most effective in treatment of patients with hereditary pancreatitis [10, 11]. TPIAT is actively used in pediatrics, as the surgery is indicated as early as possible, before the gland fibrosis, which significantly affects the islets’ dosage and quality. The vast majority of foreign medical centers demonstrate zero mortality after surgery; general analysis shows that it does not exceed 1% [12]. The results of such interventions are assessed by changes in quality of life, including elimination of pain and decrease in the need for opioids. Moreover, an important aspect of TPIAT is prevention of hypoglycemia [13]. Insulin independence, though, is not a goal in itself.

Despite the current tendency to increase the number of such surgical interventions abroad, this is not typical in Russia. This study demonstrates the earliest experience of total excision of the pancreas as a source of persistent pain with subsequent autotransplantation of the IL to treat hereditary CP.

**The aim of the study** was to assess the effectiveness of total pancreatectomy with subsequent restoration of glucose tolerance in treatment of patients with chronic genetically determined pain pancreatitis.

## Materials and Methods

The study was approved by the local ethics committee of the Privolzhsky Research Medical University (protocol No.10 dated June 24, 2022) and was conducted in accordance with the Declaration of Helsinki. Patients signed informed voluntary consent to total pancreatectomy and subsequent IL autotransplantation.

The study was performed in several stages as provided below.

### Patient examination

At this stage, a complete examination was conducted, including general clinical and laboratory analyses and investigations, genetic tests, determination of basal insulin and C-peptide, as well as glucose tolerance test.

### Surgical intervention

Pancreatectomy was conducted in the form of pylorus-preserving total pancreatoduodenectomy, because the goal was to remove pancreatic parenchyma completely, which does not provide for an intact duodenum due to impaired blood supply. Pancreatectomy was specifically aimed at maximum long-term preservation of the gland’s blood supply during the surgery. The arteries were transected immediately before the gland removal. The surgery was completed with a regular one-loop reconstruction with drains. The back table procedure was performed in parallel.

### Back table procedure

**Figure 1.**
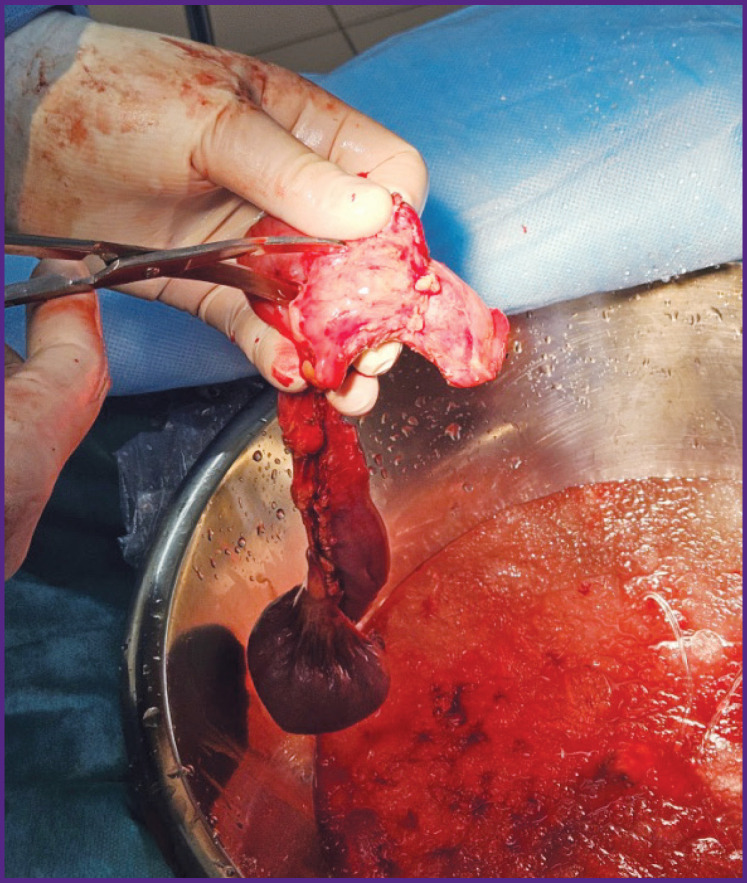
Excision of the duodenum from the pancreas

**Figure 2.**
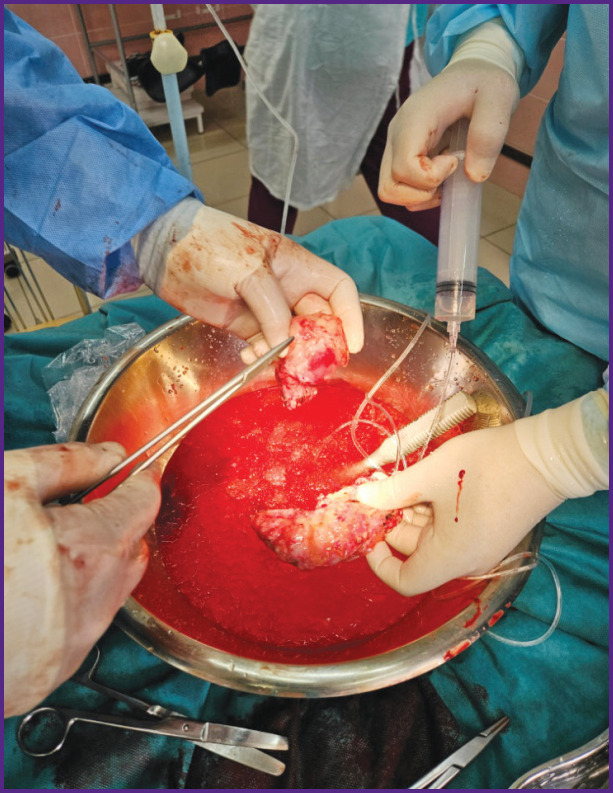
Introduction of Custodiol into the proximal and distal parts of the pancreas through catheters

The pancreatoduodenal complex was placed into a container with cold saline (+4ºС) with ice. The duodenum was separated from the pancreas (Figure 1); a small part of the gland was sent to histological analysis. Then the pancreas was transected along the isthmus. The pancreatic duct was visualized, and polyvinyl chloride catheters were fixed in the proximal and distal parts of the duct. Custodiol solution was injected into the catheter under pressure (Figure 2). After washing, the gland was placed in a sterile transportation thermal container with Custodiol solution. The thermal container with the gland was delivered to the cell laboratory in the shortest time possible.

### Cell examination. Islet isolation

IL isolation was conducted in accordance with the previously described protocol [14] with minor modifications. The isolated islets were kept in a low-glucose RPMI culture medium (Gibco, UK) supplemented with L-glutamine (0.58 mg/ml; PanEco, Russia), 10% autologous human serum, and an antibiotic and antimycotic drug (Antibiotic-Antimycotic X100; Thermo Scientific, USA) at 37°C and 5% CO2. Before transplantation, the islets were washed from the culture medium and placed into an infusion bag for delivery to the clinic.

Dithizone staining was used to confirm the IL origin of the purified cells and to assess the purity of the finally obtained preparation (20 μl of a freshly prepared 1% dithizone solution was added to 0.5 ml of the IL suspension). The IL purity was visualized using the Leica DM2500 microscope (Leica Microsystems, Germany) together with calculation of the percentage of stained IL relative to the total number of cell clusters.

Viability of the isolated islet cells was assessed by staining with trypan blue (200 μl of 0.4% trypan blue solution was added to 800 μl of the IL suspension) using the Leica DM2500 microscope (Leica Microsystems, Germany) together with calculation of the percentage of unstained IL cells relative to all cells.

Assessment of bacterial contamination was conducted by sampling the IL cell suspension (1 ml) and its transfer to the bacteriological laboratory for analysis.

### Histological examination

A pancreatic biopsy sample (~1 cm^3^) was fixed in formalin and embedded in paraffin. Pancreatic tissue sections of 7 μm thick were stained with hematoxylin and eosin in line with the standard protocol.

Immunofluorescence staining was performed on 4 μm thick sections. Immunofluorescence double staining was conducted using primary monoclonal antibodies to glucagon (1:100; Invitrogen, USA) and monoclonal antibodies to insulin (1:100; Invitrogen, USA) during 12 h at 4°C. Cell nuclei were counterstained with DAPI (1:1000; BioLegend, USA) in line with the manufacturer’s protocol. Each endocrine expressing cell was visualized using the LSM 880 confocal microscope (Carl Zeiss, Germany) and analyzed using the ImageJ 1.43u software (NIH, USA). Data are shown as a mean value ± SEM reflecting the variance within islets for each sample.

### Autotransplantation of islets

Various techniques were used to introduce the islets into the portal vein. The common technique required direct access to the portal vein with continuous direct monitoring of the portal pressure. No increase in pressure over 16 mm Hg was seen during the introduction. In clinical case 1, the X-ray surgical technique was used to introduce the islets through percutaneous and transhepatic access to the portal vein; in clinical case 2, islets were introduced into the portal bloodstream intraoperatively by implanting a catheter into the portal vein through its branch. In order to prevent activation of blood coagulation and the complement system and the associated damage to the islets the IL suspension invasion into the portal bloodstream was preceded by introduction of heparin at a rate of 50 IU/kg of body weight [15].

### Endocrinology

To assess endocrine function before surgery, a glucose tolerance test (GTT) was conducted, and basal insulin and C-peptide secretion levels were analyzed. During hospitalization, continuous glucose monitoring (CGM) using the FreeStyle Libre 2 system (Abbott, France) was conducted. Intraoperatively and in the early postoperative period, glycemia was monitored each hour by means of CGM, as well as using a glucometer; plasma glucose analysis was performed every 4–6 h. If glycemic level was above 8 mmol/L, insulin therapy was initiated. During the first and second stages of surgical intervention (pancreatectomy and IL introduction), as well as in the early postoperative period, continuous intravenous insulin infusion (CIII) was performed with an infusion pump in line with the protocol proposed by Forlenza et al. [16], as well as the protocol of Lebedeva and Vishnevsky [17]. The target glycemic values were 5.6–6.7 mmol/L. 4–6 days after surgery, patients started subcutaneous basal-bolus insulin therapy. To assess the functional state of the implanted islets, basal C-peptide was analyzed.

After introduction of islets into the portal vein, an immediate mediated inflammatory reaction in the blood was seen, as a result of which some of the transplanted islets died [14]. To minimize their loss, immunosuppressive therapy with anti-inflammatory drugs was conducted: a TNF alpha inhibitor (etanercept, 50 mg intravenously 1 h before transplantation, then 25 mg subcutaneously on days 3, 7, and 10 after transplantation) and an IL-1 receptor antagonist (anakinra, 100 mg subcutaneously on the day of transplantation and then 100 mg subcutaneously during 7 days).

### Pain assessment

Pain and quality of life were assessed before and after the TPIAT procedure. For this purpose, patients were asked to fill in the following questionnaires twice: Visual Analogue Scale (VAS); McGill Pain Questionnaire, which assessed the general pain rating index (gPRI), sensory PRI (sPRI), affective PRI (aPRI), evaluative PRI (ePRI), and selected descriptor number index (SDNI); Brief Pain Questionnaire with assessment of pain severity index (PSI) and pain interference index (PII); as well as the SF-36 quality of life questionnaire to study parameters of physical functioning (PF); role limitations due to physical health (RP); bodily pain (BP); general health (GH); vitality (VT); social functioning (SF); role limitations due to emotional problems (RE); mental health (MH); total indicators of physical (PHs) and mental (MHs) health.

## Results

### Clinical case 1

**Figure 3.**
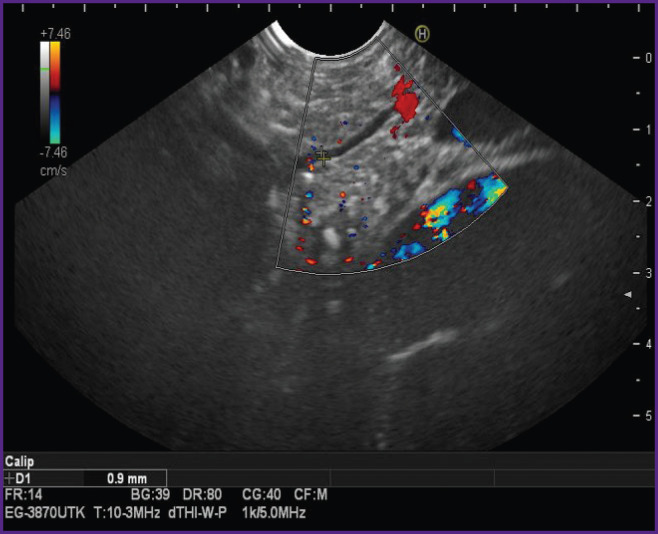
Endoscopic ultrasound examination of the pancreas: alcifications are detected in the parenchyma

**Figure 4.**
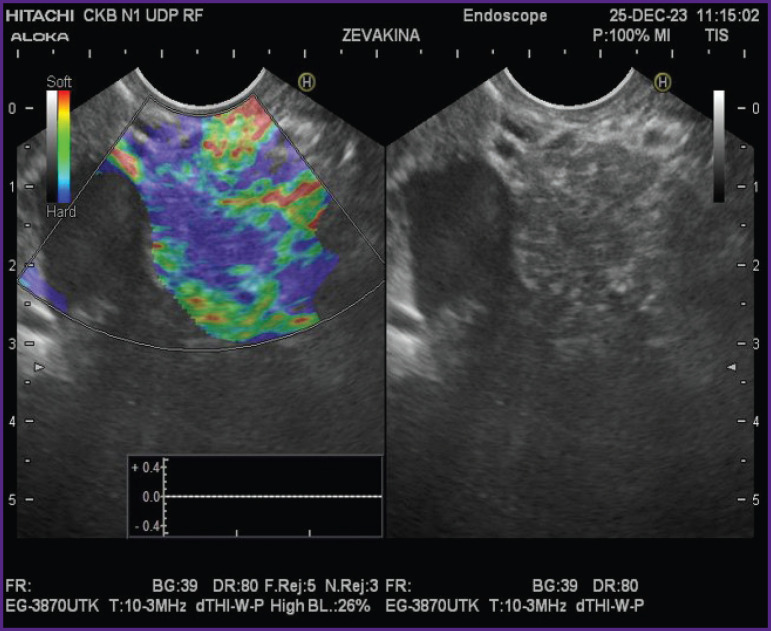
Endoscopic ultrasound examination, elastometry of the pancreas: wide areas of parenchymal fibrosis are seen

A 19-year-old female patient complained of girdle-like pain in the epigastrium, nausea, and constipation. Medical history of chronic pancreatitis for 10 years. The first attack with inpatient treatment was in 2015, after which she had repeated hospitalizations due to severe pain (every 3 months). The results of spiral computed tomography and endoscopic ultrasound provided as follows: calcifications in the fibrous parenchyma of the pancreas, the main pancreatic duct was slightly dilated — up to 3–4 mm (Figures 3, 4). In 2023, stenting of the main and accessory pancreatic ducts was conducted several times; after interventions, according to the patient, the pain intensified.

In 2022, the patient lost about 12 kg of body weight in six months, which caused secondary amenorrhea; in 2023, she partially regained the lost weight.

In December 2023, due to severe pain, the patient underwent a diagnostic laparoscopy (suspected perforation of a hollow organ). Additional genetic testing revealed a homozygous mutation c.101A>G(p/Asn34Ser) of the *SPINK1* gene.

Preoperative examination of the endocrine function of the pancreas did not identify any glycemic disorders. According to the GTT results, fasting glycemia was 5.2 mmol/L, 1 h after the glucose load — 6.91 mmol/L, 2 h after the glucose load — 3.32 mmol/L. Basal insulin and C-peptide levels were also within normal range — 6.65 μU/ml and 1.42 ng/ml, respectively. Glycated hemoglobin before surgery was 4.19%. The HOMA1 index was 48.9 and corresponded to normal β-cell function; HOMA1-IR was 1.54 and corresponded to normal sensitivity to insulin. At that, the QUICKI index of 0.36 indicated early insulin resistance.

Instrumental research techniques (CT of the chest organs, EGD, ultrasound, ECG, CT of the abdominal cavity with intravenous contrast) did not reveal any abnormalities.

The patient was enrolled to the study on April 5, 2024 with the following diagnosis: hereditary calcifying pancreatitis (homozygous mutation c.101A>G of the *SPINK1* gene), clinical phenotype — recurrent acute pancreatitis.

Total pancreatoduodenectomy was conducted on October 9, 2024 in line with the protocol described above.

The rate of CIII insulin administration was 0.5 [0.2; 1.2] U/h. The daily dose of insulin in the intraoperative period was 20 [18; 22] U/day. Glycemia — 6.2 [5.4; 7.4] mmol/L. The patient had CIII during the first 6 days after surgery.

Intraportal administration of the islet suspension was performed on October 10, 2024 under combined anesthesia (immediate local anesthesia and local anesthesia with 0.5% novocaine solution, 20 ml). With ultrasound control, a puncture of the segmental branch of the portal vein of the right lobe of the liver was performed with a CHIBA 20 G needle through the 8^th^ intercostal space on the right along the anterior axillary line. Portography showed that the portal blood flow was not compromised. A hydrophilic reinforced introducer Flexof 5 Fr (COOK Medical, Ireland) was installed using a guidewire into the lumen of the portal vein, in the area of the splenomesenteric anastomosis. Heparin was administered (3500 U). Manometry in the portal vein system was conducted (the average pressure was 13–15 mm Hg). Then, during 30 min 200 ml of islet cell suspension was infused sequentially at a rate of 400 ml/h. Control manometry was performed in the portal vein basin (the pressure was 16–17 mm Hg). Control portography showed no compromise of the blood flow. For hemostasis, the puncture channel was sealed with hemostatic sponge foam. 15 min after introducer removal, ultrasound showed no hematomas or free fluid in the liver or abdominal cavity.

Postoperative period was complicated by obstructive jaundice, which was associated with the narrowing of the hepaticojejunostomy. External drainage of the bile ducts was performed under ultrasound fluoronavigation. After 4 days, the external cholangiostomy was replaced with a bipolar one. On Day 20, the patient was discharged in satisfactory condition.

### Clinical case 2

A 28-year-old female patient. Medical history of CP for 23 years. The first attack with inpatient treatment was at the age of 5, followed by the patient repeated hospitalizations up to 4 times a year. In 2022 and 2023, endoscopic retrograde cholangiopancreatography and endoscopic papillosphincterotomy were conducted with a minimal positive effect. In 2024, the patient had 4 hospitalizations with attacks of acute pancreatitis. A genetic study in the *PRSS1* gene revealed the R122H polymorphism in a heterozygous form, which is a sign of conservative therapy futility and a high risk of malignancy. The patient took Ermital up to 150,000 U/day, Duspatalin, Heptor, Razo, and Dalargin on a permanent basis. Frequent intense pain was relieved by taking analgesics and octreotide.

GTT showed that the fasting glycemia level was 5.34 mmol/L, after 2 h — 8.01 mmol/L. These glycemia levels indicate the initially impaired glucose tolerance. Preoperative insulin and C-peptide levels were within normal range — 15.64 μU/ml and 1.7 ng/ml, respectively. Glycated hemoglobin — 4.81%. The HOMA1 index before the surgery was 170, which corresponds to normal β-cell function. Insulin resistance was proved by the following: HOMA1-IR — 3.71 and QUICKI — 0.31.

The patient was enrolled to the study on September 16, 2024 with the following diagnosis: hereditary painful pancreatitis (heterozygous mutation of the *PRSS1* gene), continuously recurring disease form (7 or more attacks per year during the period from 2001 to 2024).

Endoscopic papillosphincterotomy was conducted on April 5, 2022 and November 30, 2023.

Surgical intervention was performed on September 18, 2024 (total duodenopancreatectomy in line with the technique described above). Intraportal administration of islets was conducted on September 19, 2024.

Relaparotomy was performed with removal of postoperative sutures. The branch of the middle colic vein was visualized; it was cannulated with an Fr7 catheter inserted into the lumen of the portal vein. 3500 U of heparin were administered. Manometry was performed in the portal vein (the average pressure was 13 mm Hg). Then during 30 min, sequential infusion of 200 ml of islet cell suspension was performed at a rate of 400 ml/h. Manometry in the portal vein pool was conducted every 10 min; during control manometry, the pressure was 13–15 mm Hg. The wound was sutured.

Postoperatively, gastrostasis was seen, nutritional support was adjusted. On day 14, positive dynamics in the form of complete relief of gastrostasis was noted. On day 19, the patient was discharged in a satisfactory condition.

### Endocrinology

The CIII insulin rate was 0.5 [0.2; 1.2] U/h for clinical case 1 and 1.0 [0.5; 1.5] U/h for clinical case 2. The daily insulin dose in the intraoperative period was 20 [18; 22] and 20 [18; 35] U/day, respectively. The median glycemia was 6.2 [5.4; 7.4] and 7.7 [6.0; 8.7] mmol/L. Continuous insulin infusion was performed during the initial 5–6 days. It is notable that in clinical case 1, when glycemia was monitored using the FreeStyle Libre 2 system (Abbott, France) in the intraoperative and early postoperative period, low blood sugar levels were often recorded, which was not confirmed by a study of capillary and venous blood. This forced the researchers to refuse CGM in the initial days after surgery and use a glucometer to assess glycemia. This may be due to known artifacts [18], as well as impaired peripheral circulation in the early postoperative period and, thus, be accompanied by low glycemic values in the interstitial fluid. In clinical case 2, no differences were observed between the readings of FreeStyle Libre 2 (Abbott, France) and the glucometer, which allowed to significantly reduce the use of the glucometer and effectively assess glycemic fluctuations, as well as select insulin doses using CGM.

Both patients had ketonuria (0.5 mmol/L) diagnosed on the 3^rd^ postoperative day; it was associated with malnutrition in the early postoperative period (Table 1) and was discontinued by intravenous administration of 5% glucose solution.

**T a b l e 1:** Laboratory data on endocrine function of the pancreas and insulin requirements in dynamics in patient 1 before and after TPIAT

Parameter	Before surgery	Early postoperative period	1 month after surgery	3 months after surgery	Standard level
Fasting glucose (mmol/L)	5.2	5.9 [5.3; 6.6]*	6.6 [5.5; 7.5]*	5.2 [5.1; 6.7]*	3.0–6.4
Daily glycemic level	—	6.2 [5.4; 7.4]*	6.7 [5.2; 8.2]*	5.2 [5.0; 7.2]*	—
C-peptide (ng/ml)	1.42	0.02	0.025	0.02	1.1–4.4
Insulin (μU/ml)	6.65	—	—	—	2.6–24.9
Glycated hemoglobin (%)	4.19	—	—	5.1	0–6
Insulin requirement (U/kg)	—	0.19–0.42	0.39–0.47	0.42–0.47	—

* Data are shown as Me [Q1; Q3].

**Figure 5.**
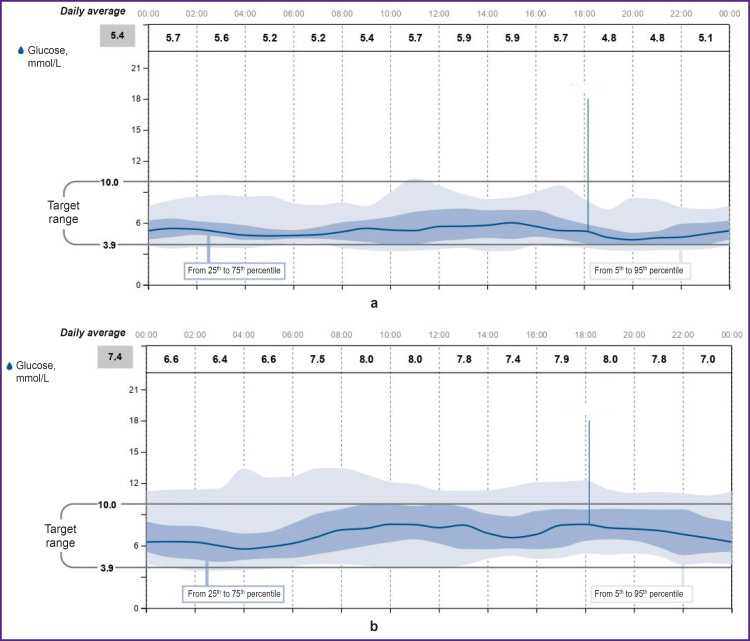
Continuous glucose monitoring with the FreeStyle Libre 2 system (Abbott, France) during the first 14 days of inpatient treatment after TPIAT: (a) clinical case 1; (b) clinical case 2

**Figure 6.**
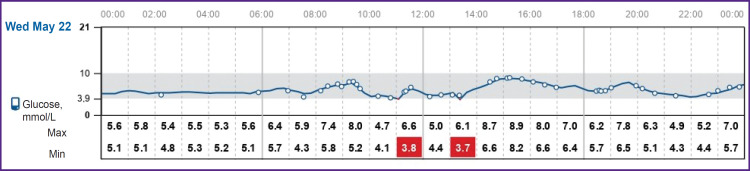
Diagram of continuous glucose monitoring with the FreeStyle Libre 2 system (Abbott, France) on day 14 after surgery of patient 1

After the patients were transferred to basal-bolus insulin therapy, the initial insulin requirement for maintaining normoglycemia was 0.19 and 0.28 U/kg for clinical cases 1 and 2, respectively. With a less strict diet in patient 1, the insulin requirement increased to 0.42 and 0.50 U/kg during the 1^st^ month, and after 6 months it was 0.47–0.50 U/kg. The doses of bolus insulin injections during the morning meal were calculated as follows: 1 BU: 1.5 U, whereas during lunch and dinner — 1 BU: 1 U. Both patients were trained in insulin dose selection and adjustment. Diagrams of continuous glucose monitoring with the FreeStyle Libre 2 system (Abbott, France) during inpatient treatment are shown in Figures 5 and 6. In hospital, the average glucose level in clinical case 1 was 5.4 mmol/L, in clinical case 2 — 7.4 mmol/L. The time in the target range was 85 and 79%, respectively; hypoglycemia in the range of 3.0–3.8 mmol/L — 4%. There was no hypoglycemia below 3 mmol/L noted.

One month after surgery self-control assessment demonstrated that fasting glycemia was 6.6 [5.5; 7.5] and 7.7 [5.0; 8.8] mmol/L, in 2 h after meal it reached 5.2 [5.0; 7.2] and 7.9 [6.4; 9.4] mmol/L for patients 1 and 2, respectively. However, according to FreeStyle Libre 2 (Abbott, France), patient 2 had an average glycemia level of 10.3 mmol/L 6 months after TPIAT, whereas the time in the target range was 47%. This fact may indicate the need to repeat patient training, including in a diabetes school, and also is a basis to consider prescribing insulin pump therapy.

The basal C-peptide levels in dynamics after the first and second stages of surgery in both clinical cases remained below the standard and amounted to 0.01 and 0.02 ng/ml, respectively. The data on carbohydrate metabolism and endocrine function parameters of the pancreas of patient 1 in dynamics are shown in Table 1.

A corresponding enzyme replacement therapy with Creon in a total dose of up to 250,000 and 200,000 U/day was selected for patients 1 and 2, respectively.

### Pain assessment

Based on the analysis of answers to questionnaires used to assess the dynamics of pain and quality of life, it was noted that both patients rated their pain as severe before the surgery (10 points according to VAS); in addition, the gPRI, sPRI, aPRI, ePRI indices according to the McGill Pain Questionnaire, as well as the PSI and PII on the Brief Pain Questionnaire were high. One month after the surgery, the patients complained of virtually no pain and noted an improvement in their quality of life (Table 2); all the parameters of the VAS questionnaire, the Brief Pain Questionnaire, and the McGill questionnaire decreased significantly. Assessment of the quality of life using the SF-36 questionnaire highlighted the improvement in the values of physical functioning, general health, vitality, social functioning, and mental health in the dynamics after the surgery.

**T a b l e 2:** Results of questionnaires assessing patients’ pain and quality of life before and after TPIAT

Questionnaire	Questionnaire tag	Patient 1		Patient 2	
		Before surgery	After surgery	Before surgery	After surgery
VAS		10	1	10	0
McGill Pain Questionnaire	SDNI	20	3	11	5
	PRI	60	5	22	7
	gPRI	37	4	10	6
	aPRI	18	0	8	0
	ePRI	5	1	4	1
Brief Pain Questionnaire	PSI	2.5	1	8.5	2
	PII	33	2	7.6	0
SF-36	PF	90	90	25	60
	RP	0	25	75	75
	BP	41	74	22	94
	GH	25	82	50	65
	VT	25	75	10	40
	SF	37.5	87.5	50	62.5
	RE	0	33.3	67	67
	MH	36	88	64	92
	PHs	41.3	48	26.23	41.8
	MHs	26.7	49.9	43.8	50.5

### Cell examination. Islet isolation

**Figure 7.**
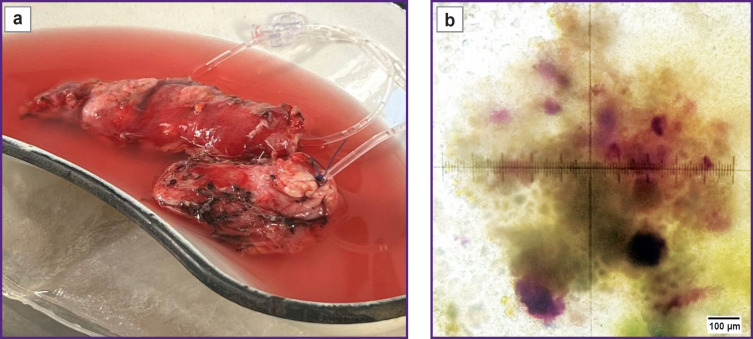
Procedure of enzymatic degradation of pancreas: (a) perfused pancreas; (b) pancreatic tissue; enzymatic degradation with collagenase; islets of Langerhans are stained in crimson (dithizone staining)

After total pancreatectomy by means of the described technique, human islet cells free of acinar tissue were successfully isolated from the pancreases (141 g for patient 1; 80 g for patient 2) (Figure 7).

For patient 1, the volume of cell suspension was 0.5 ml, containing 5,000 islet equivalents (IEQ); for patient 2 — 2 ml of cell suspension containing 42,000 IEQ. The final amount of IEQ for patient 2 was higher than for patient 1, which may be due to less pronounced fibrosis of the pancreas. The number of equivalents was assessed based on the number and size of the islets (Table 3). This is an important indicator for assessment of the quality and quantity of the isolated IL, which are involved in production of insulin and other hormones that regulate blood glucose levels.

**T a b l e 3:** Formula for calculation of the number of islet equivalents

Islet diameter (μm)	IEQ, conversion factor
50–100	×0.167
101–150	×0.648
151–200	×1.685
201–250	×3.5
251–300	×6.315
301–350	×10.352
>350	×15.833

**Figure 8.**
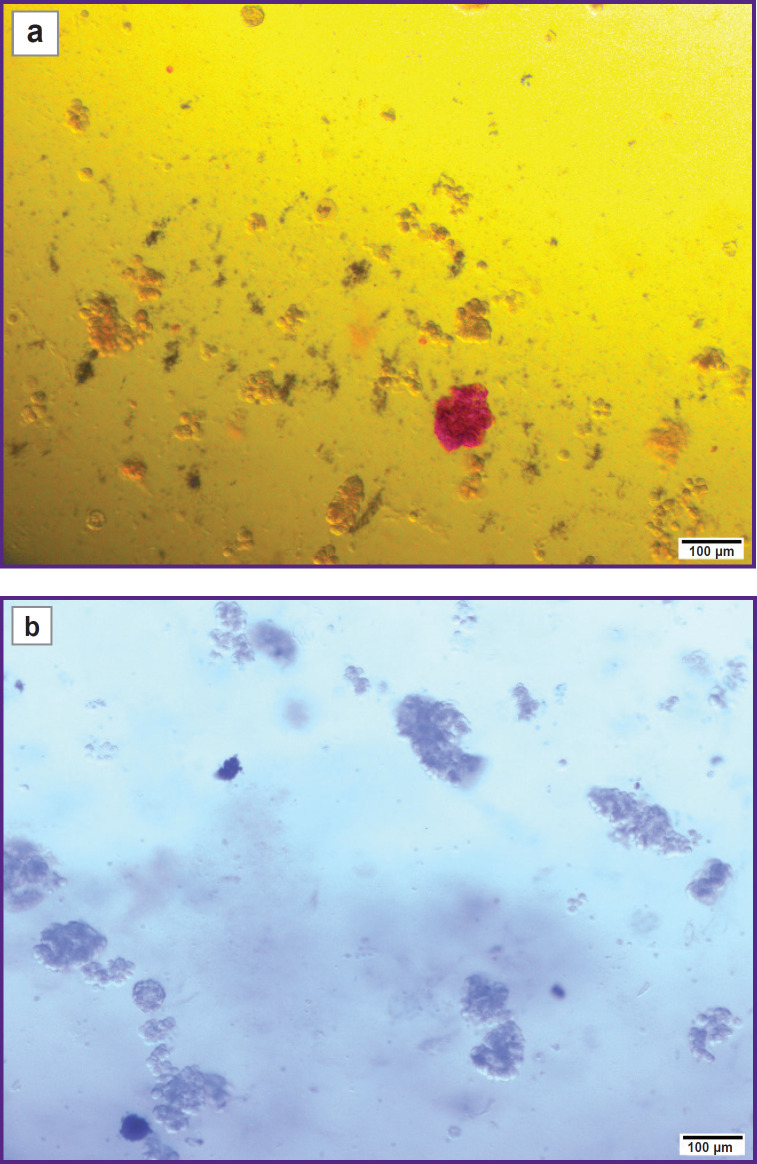
Isolated islets of Langerhans: (a) assessment of purity, islets are stained in crimson with dithizone; (b) assessment of viability, dead cells are stained in dark blue with trypan blue

The final purity of the IL suspension was 15%, which indicates presence of other cell components in the suspension (Figure 8 (a)). However, high survival rate (Figure 8 (b)) of the IL after isolation, which was 95% in clinical case 1 and 85% in clinical case 2, demonstrated that the extraction technique was effective and minimally traumatic for the cells (Table 4).

**T a b l e 4:** Comparative characteristics of the isolated islets

Parameter	Patient 1	Patient 2
Duration of chronic pancreatitis (years)	10	23
Cell volume (ml)	0.5	2
Total number of islet equivalents	5000	42,000
Viability (%)	95	85
Purity (%)	15	15

Bacteriological analysis confirmed sterility of the isolated islets, which is a critically important factor for their further clinical use.

### Histological examination

**Figure 9.**
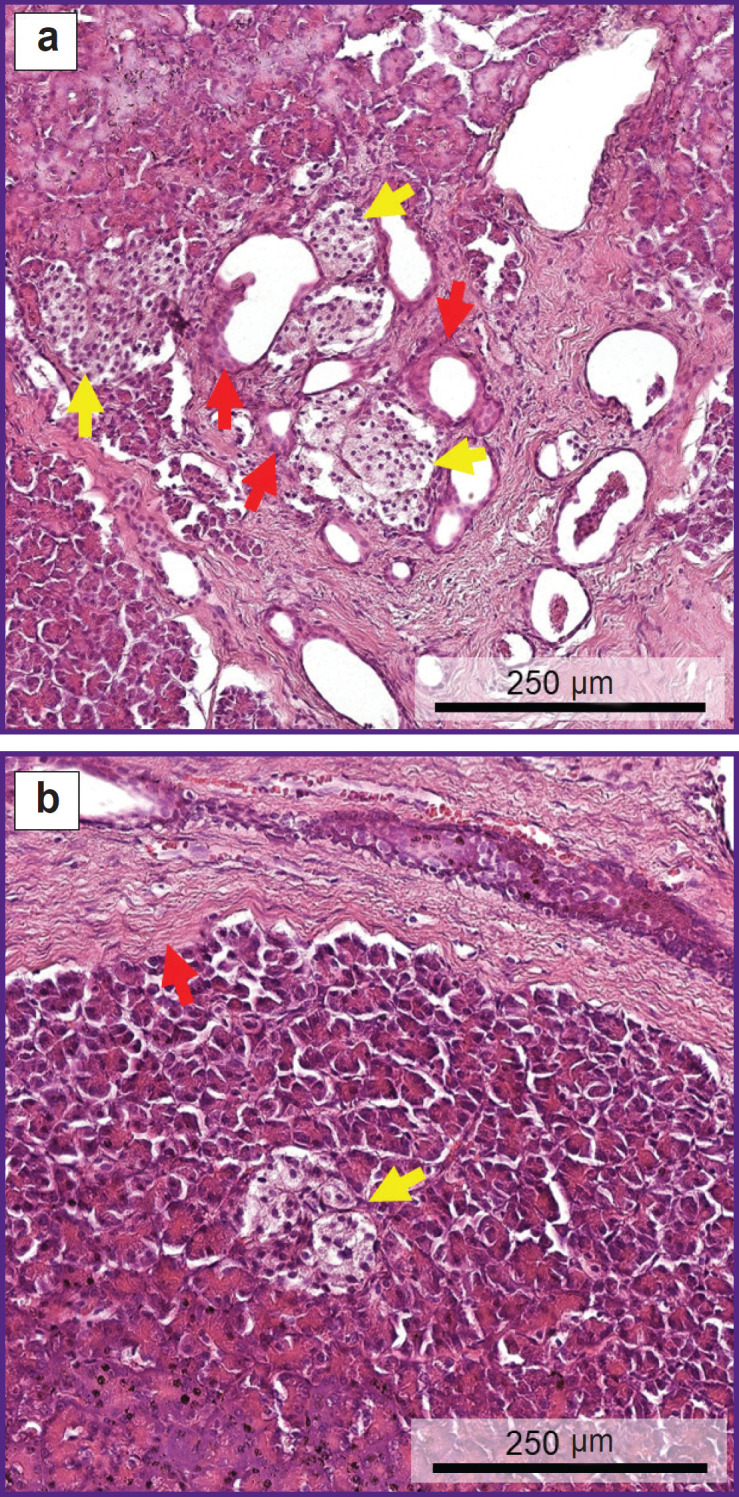
Representative histological images of pancreatic tissue (staining with hematoxylin and eosin): (a) area of centrilobular fibrosis with atrophy and acinarductal metaplasia (*red arrows*); some cells in metaplastic ducts contain eosinophilic cytoplasmic granules typical of acinar cells; loss of acinar tissue precedes loss of islets, which are located in groups of various size in fibrosis (*yellow arrows*); (b) islet (*yellow arrow*) in an area of pancreas with preserved acinar tissue and perilobular fibrosis (*red arrow*)

To assess the quality of the pancreas, a histological examination was conducted which confirmed CP. The pancreas was depicted by sample areas with pronounced interlobular (perilobular) and intralobular fibrosis. Significant loss of acinar tissue was noted as a result of atrophy and acinarductal metaplasia (Figure 9 (a)). Intralobular ducts were deformed, the epithelium of the ducts was atrophic, some ducts contained eosinophilic protein plugs in the lumen. Fibrous areas had residual elements in the form of IL, blood vessels, and nerve bundles. Residual islets form groups of various sizes and, on average, contain smaller cells than normal islets, which may indicate proliferation of A-cells and PP-cells in CP [19]. Areas with intact acinar tissue (Figure 9 (b)) showed a small number of IL with a structure almost typical of normal. There was also a moderate diffuse chronic inflammatory infiltrate.

**Figure 10.**
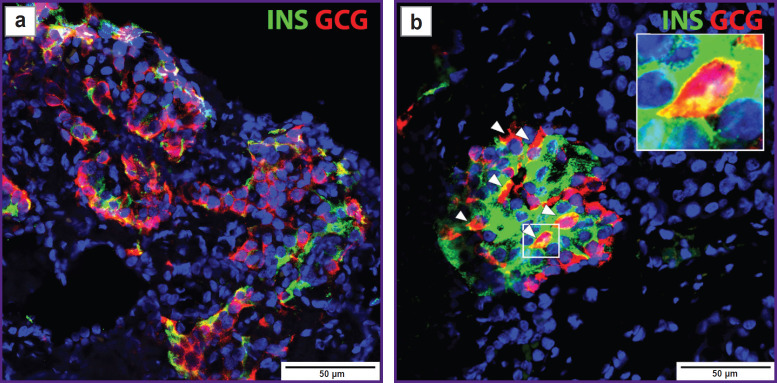
Immunohistochemical а b staining of insulin-expressing cells (*green*) and glucagon (*red*) as well as cell nuclei (*blue*): (a) high concentration of islets in the affected area of the pancreas; (b) extensive cosecretion of hormones specific for α- and β-cells (arrows point at bihormonal cells)

Immunofluorescence staining of the pancreas revealed 16.35±2.93% endocrine cells, mainly in 4/10 islets, which were bihormonal, coexpressing both insulin and glucagon (Figure 10).

## Discussion

Total pancreatectomy with islet autotransplantation (TPIAT) in CP is conducted in many medical centers around the world. There is a tendency to form highvolume medical centers to increase patient flows and improve results [20, 21]. TPIAT is recommended for chronic pain pancreatitis, as it is quite safe; even in case of failure to achieve insulin independence, it significantly decreases the risk of complications from severe hyperglycemic conditions and greatly improves the quality of life [22]. In CP, obtaining islets and posttransplant endocrine function depend on the degree of fibrosis and previous pancreatic surgeries [23]. Therefore, the worldwide trend to shift the technology to pediatrics seems logical.

The patients in this study had genetically determined severe pancreatitis with the likelihood of pancreatic cancer development as well as the futility of drug treatment. Homozygous mutation of the *SPINK1* gene and heterozygous mutation of the *PRSS1* gene are the most widespread. Clinically, both patients had the disease characterized by early onset, unusually severe and persistent course with frequent repeated attacks, which became a basis for an in-depth study for genetic mutations.

The previously conducted IL isolations in the author’s earlier studies [14] showed their functional viability.

IL autotransplantation was conducted both by means of minimally invasive and upfront surgery. The literature does not indicate any preferred technology for it. Apparently, the choice of a technique is rather a tradition of a clinic.

After TPIAT, both patients noted a significant decrease in the pain intensity. Minor pain persisted during the first month, which was often associated with surgical intervention and abdominal discomfort at selection of adequate doses of enzyme replacement therapy. According to the patients, they did not take any analgesics after the surgery, which is comparable with the results of foreign studies [24]. The major achievement, according to the results of the SF-36 questionnaire, is the registered improvement in the quality of life (consistent with other researchers’ data) [21]. At that, patient 1 showed an improvement in all parameters, and this was related to the general health, vitality, and mental health, whereas patient 2 had these parameters increased, but to a lesser extent, and physical functioning did not change after the surgery. This fact may be attributed to a longer CP course in clinical case 2.

Hereditary/genetic pancreatitis (HGP) is a specific type of CP that is an indication for TPIAT. It is acknowledged that patients with hereditary pancreatitis or *PRSS1* gene mutations face an increased risk of pancreatic cancer. In such patients, TPIAT is aimed to remove the pancreas as a potential source of ductal adenocarcinoma.

Hereditary/genetic pancreatitis is caused by a mutation in the gene that encodes cationic trypsinogen (protease serine 1, or *PRSS1*) and is mapped to 7q35 on the long arm of human chromosome 7. Pathogenic mutations in *PRSS1* are associated with an over 80% chance of recurrent acute and/or chronic pancreatitis, as well as an exceptionally high lifetime risk of pancreatic ductal adenocarcinoma (estimated at 40%). TPIAT is indicated to young patients (often <10 years) showing the first symptoms of the disease. Hence, in patients with HGP, TPIAT is aimed not only to relieve pain and improve quality of life, but also to reduce the risk of pancreatic cancer.

A direct correlation between the duration of HGP and clinical diabetes development was found. When comparing patients with HGP and patients without known genetic mutations, the first group demonstrated more pronounced fibrotic changes in pancreatic tissue, lower islet mass, and a significantly lower likelihood of achieving insulin independence in the future compared to the group with non-genetic CP options. A study by Chinnakotla et al. [25], that enrolled 80 patients with HGP, showed that TPIAT in patients with HGP ensured long-term pain relief (90%) and preservation of β-cell function.

Patients with HGP and high risk of pancreatic cancer should be considered for TPIAT as earlier as possible, before pancreatic inflammation leads to a high degree of parenchymal fibrosis and loss of IL function. Therefore, such patients are usually younger compared to patients undergoing TPIAT for other indications. In the study of 64 patients with HGP, Bellin et al. [26] showed that postoperative outcome was adversely affected by older age and disease length. In particular, islet mass was lower and the risk of diabetes was higher in older patients with a long history of pancreatitis. This should be taken into account at consultations to a subgroup of potential TPIAT recipients.

Development of diabetes in CP is associated with the IL destruction under the influence of inflammatory processes in the pancreas. The level of insulin production after TPIAT usually correlates with the mass of transplanted islets (Table 5) and with preoperative analyses of metabolic function and HbA1c. Effective transplantation is mainly reported when over 5000 IEQ/kg are transplanted [23, 27–28], but there are examples of achieving insulin independence after transplantation of less than 1000 IEQ/kg [29]. We performed 2 TPIAT procedures, with 83 IEQ/kg transplanted in clinical case 1 and 553 IEQ/kg in clinical case 2. Low number of transplanted ILs may explain the negative results of graft functioning. The information on dependence of the clinical effectiveness of transplanted islet function on their number which was collected and systematized in the study is presented in Table 5.

**T a b l e 5:** Gradation of the transplant efficiency (insulin independence) dependence on the number of introduced islet equivalents (IEQ)

Parameter	<2500 IEQ/kg	2500–5000 IEQ/kg	>5000 IEQ/kg
Efficiency of dissection (%)	23.0	49.1	27.9
Insulin independence during a year (%)	32	79	86
Insulin independence during 3 years (%)	12–15	20–35	65–72
Complete insulin independence (%)	6.7	34.6	45.7
Partial insulin independence (%)	53.5	65.4	54.3
Inefficiency (%)	40	0	0

However, even the correlation between the mass of transplanted islets and the success of transplantation is imperfect. Beamish et al. [23] suggest that differences in the IL function before and after TPIAT may be associated with changes in the identity of β-cells in patients with CP. As in the considered cases, the researchers found an increased content of bihormonal cells. Moreover, Beamish et al. conducted additional studies and showed a possible correlation of dedifferentiated endocrine cells in patients with long-term CP and a decrease in the autotransplantation effectiveness. The prevalence of bihormonal cells does not seem to be associated with clinical parameters and pretransplant glucose levels. However, preoperative increases in proinsulin, insulin, and proinsulin/insulin ratio in non-diabetic individuals were probably consistent with the histological data on cellular dedifferentiation. This observation, together with recently reported cases of dedifferentiation in islet allograft recipients and the relation of this phenomenon with IL isolation [30], highlights the need for further study of the dedifferentiation status of transplanted islets during TPIAT, as well as for assessment and clinical usefulness of IL autotransplantation.

In both cases, insulin resistance was identified, which can also be an obstacle to normalization of glycemia. Insulin resistance in such patients is most often of hepatic origin and occurs in long-term CP due to a decrease in the number of PP cells and, thus, a decrease in the synthesis of pancreatic polypeptide [31].

## Conclusion

The study demonstrates the first Russian experience of treatment chronic pancreatitis by total removal of the pancreas being a source of persistent pain followed by autotransplantation of the isolated islets of Langerhans. This technique is safe for patients and highly effective in pain reduction, as well as in pancreatic cancer prevention.

The analysis of possible causes of functional insufficiency of the transplanted islets in this study allows to conclude the necessity to use TPIAT earlier in genetically determined pancreatitis, when a sufficient number of functioning islets is preserved in the inflamed gland. Timely diagnosis of genetically determined pancreatitis in younger age will help to avoid long-term futile and ineffective treatment.
